# Impact of Healthcare Disparities on Time to Surgery for Pediatric Urologic Patients

**DOI:** 10.7759/cureus.25711

**Published:** 2022-06-07

**Authors:** Thomas E Schroeder, Kaeli K Samson, Ellen Kerns, Claudia Berrondo

**Affiliations:** 1 Urology, University of Nebraska Medical Center, Omaha, USA; 2 Biostatistics, University of Nebraska Medical Center, Omaha, USA; 3 Pediatrics, University of Nebraska Medical Center, Omaha, USA; 4 Pediatric Urology, Children's Hospital & Medical Center, Omaha, USA

**Keywords:** population-based study, time to surgery, sociodemographic differences, pediatric urology, healthcare inequality

## Abstract

Introduction

Healthcare disparities are differences in health outcomes reflecting social inequalities. We aim to identify healthcare disparities in pediatric urologic patients by analyzing the time from surgical scheduling to completion of procedure at a single center and identify variables associated with increased time to surgery.

Materials and methods

We reviewed all patients aged 0-18 years who underwent surgery with one of three pediatric urologists at our institution from January 1, 2018, to December 31, 2019. We collected or calculated variables including age, sex, race, ethnicity, caregivers’ primary language, insurance status, zip code, median distance to hospital, clinic visit date, and time to surgery (calculated as days between surgery request and date of surgery). Data analysis included bivariate analysis and linear regression with all variables of interest presented with 95% confidence intervals (CIs), where log-transformed time to surgery was the outcome. Because the practice at our institution is to delay elective surgeries until after six months of age, we excluded patients who were less than six months of age at the time of surgery request date.

Results

A total of 697 patients were included in the final analysis. Patients’ caregivers who spoke languages other than English or Spanish had a lower model-adjusted mean log-days to surgery (−0.44; 95% CI: −0.85, −0.03) relative to English-speaking caregivers. Uninsured patients had increased time to surgery compared to Medicaid patients (0.28; 95% CI: 0.03, 0.53). Income was also associated with increased time to surgery, meaning patients from higher-income backgrounds had a longer time to surgery (0.04; 95% CI: 0.00, 0.08).

Conclusions

In our patient population, primary language spoken and insurance status were associated with increases in time from initial evaluation to surgical intervention among pediatric patients undergoing urologic surgery. Additional research is needed to better understand variations in access to pediatric urologic surgery.

## Introduction

Healthcare disparities are differences in health outcomes between groups that reflect social inequalities and have been widely documented over the last several decades in various forms throughout the United States [1]. Individuals of minority racial and ethnic groups are often identified as receiving disparate care in several areas of both primary care and specialty healthcare services [2-5]. However, access to healthcare is complex and dependent on multiple factors. Some studies have documented that socioeconomic status and income [5,6], distance to hospitals [7], insurance status [4,8-10], and type of hospital and procedure [5,11,12] contribute to healthcare disparities.

Although many studies investigating healthcare disparities in pediatric populations have focused on specific conditions including asthma [3,6] and otitis media [12], to our knowledge, there are few studies evaluating healthcare disparities and access for patients needing pediatric urologic surgeries [13-16], with the majority focusing on ureteropelvic junction obstructions. To address inequalities in our patient population, we must first understand whether inequalities exist, and define those inequalities.

This study aimed to identify healthcare disparities among pediatric patients undergoing urologic surgery by analyzing the time from surgical scheduling to the completion of the procedure at a single center. We hypothesized that healthcare disparities exist in this population.

## Materials and methods

Data collection

With institutional review board (IRB) approval (protocol #315-20-EP), we performed a retrospective chart review using electronic medical records of patients aged 0-18 years who underwent elective surgery with three pediatric urologists at our institution from January 1, 2018, to December 31, 2019. The clinical visits and surgeries were performed at the regional children’s hospital, associated outreach clinics, and outpatient surgical center.

Demographic and clinical data extracted from the medical records for the analysis included medical record number, date of birth, age at the time of surgery request, sex, race, ethnicity, caregivers’ primary language, insurance status, zip code, median distance to hospital, clinic visit date, surgery request date, date of surgery, and type of surgery performed.

We calculated the median drive time from the patient’s zip code to the clinic in minutes using isochrones (drive time zones surrounding a location) [17]. Isochrones of five-minute increments up to 300 minutes were generated around the clinic using the R package “osrmIsochrone” [18], and patients' home zip codes were categorized as to which zone they fell within. If more than one, the median across all drive time zones that zip code fell in was chosen. Patients' zip codes falling outside the 300-minute isochrone were assigned > 300 minutes. Due to the large variation in travel time among patients, the time to the clinic was divided into four separate categories: less than or equal to 30 minutes, 31-45 minutes, 46-106 minutes, and greater than 106 minutes. The median household income for the patient’s zip code was retrieved from the American Community Survey (ACS) [19]. “Surgery request date” was defined as the date that the urologist sent the surgery-scheduling request to the surgery scheduler. The variable for “time to surgery” was defined as the time (in days) between the surgery request date and the date the surgery was performed. Surgeries were categorized as penile surgery, testicular surgery, hydrocele/hernia, endourology, ureteral reconstruction, and others. Categories were developed by an expert review and consolidated to ensure sufficient power for statistical analysis. For patients who underwent multiple surgeries, only their first surgery was included in the analysis. Because the practice at our institution is to delay elective surgeries until after six months of age, we excluded children younger than six months of age at the time of surgery scheduling. Patients who underwent same-day surgery were excluded in an attempt to remove emergent surgeries as a possible confounder.

Statistical methods

Descriptive statistics are presented as counts and percentages or medians and interquartile ranges (IQRs). Differences in time to surgery were assessed using Wilcoxon rank-sum tests for dichotomous variables or Kruskal-Wallis tests for categorical variables with three or more groups. Significant Kruskal-Wallis tests were followed up with all pairwise comparisons of groups using Wilcoxon rank-sum tests, and the associated p-values were Bonferroni adjusted. Associations between the time to surgery variable and continuous variables were assessed using Spearman correlations. For modeling, the outcome variable time to surgery was log-transformed and then all variables of interest were included in a linear regression model. All 95% confidence intervals (CIs) for model estimates were visualized in a forest plot; CIs for variables with more than two subgroups were adjusted using the Tukey-Kramer method. For more intuitive interpretations, model-adjusted means of log-days to surgery and associated 95% CIs were computed for each subgroup, or for two subgroups with a one-unit difference for continuous, linearly modeled variables (e.g. one vs. two-year-old patients and patients with median family incomes of $60,000 vs. $70,000, as median income was represented in $10,000). Model-adjusted means of log days to surgery and CIs were then exponentiated to back-transform them into geometric means, which have the original unit of days. P-values for post-hoc pairwise comparisons were Tukey-Kramer adjusted for significant model variables with more than two subgroups. All analyses were performed using SAS software version 9.4 (SAS Institute Inc., Cary, NC).

## Results

Patient characteristics

A total of 697 patients met the inclusion criteria. Of our original sample size of 1011 patients, we excluded 238 patients who were younger than six months of age at the time of surgery scheduling, 75 patients who were identified as urologic emergencies and received same-day surgical intervention, and one patient with a complex reconstructive surgery, given the small sample size of this surgical subgroup.

Patients' age at the time of surgery scheduling ranged from six months to 18.4 years, with a median age of 3.7 years (IQR: 1.2, 8.5). Of patients, 92% were males and 8% were females. The majority of patients were White (64.8%) and English-speaking (88.9%). Nearly half (45.4%) of the patients had commercial insurance. Penile surgery was the most performed procedure composing 46.2% of cases (Table 1).

**Table 1:** Patient demographics. The distance is measured as time from home zip code to hospital in minutes.

Characteristics	Patients N (%)
Gender	
Female	55 (7.9)
Male	642 (92.1)
Race/ethnicity	
Asian	30 (4.3)
Black	65 (9.3)
Hispanic	92 (13.2)
White	451 (64.8)
Other	58 (8.3)
Language	
English	619 (88.9)
Spanish	39 (5.6)
Other	38 (5.5)
Distance	
<=30 mins	200 (28.7)
31-45 mins	147 (21.1)
46-105 mins	171 (24.5)
>=106 mins	179 (25.7)
Insurance	
Commercial	316 (45.4)
Medicaid	265 (38)
Uninsured/self-pay	116 (16.6)
Type of surgery	
Endourology	132 (18.9)
Hydrocele/hernia repair	83 (11.9)
Penile surgery	322 (46.2)
Testicular surgery	121 (17.4)
Ureteral reconstruction	9 (1.3)
Other	30 (4.3)

Bivariate analysis

There was no significant association between time to surgery and age (Rho = −0.01; p = 0.88) or gender (p = 0.25). White patients tended to have a longer median time to surgery (32 days; IQR: 16, 55) compared to Black patients (19 days; IQR: 10, 43) (p < 0.04). Patients with English-speaking caregivers tended to have longer times to surgery (32 days; IQR: 15, 53) compared to patients whose caregivers spoke languages other than English or Spanish (17 days; IQR: 9, 28) (p < 0.001; Table 2).

**Table 2:** Bivariate analysis investigating time to surgery. P-values are from Kruskal-Wallis (KW) tests unless otherwise indicated. † Spearman correlation. †† Wilcoxon rank-sum test. Bonferroni-adjusted post-hoc pairwise comparisons when the KW test was significant. * Per Bonferroni-adjusted post-hoc pairwise comparisons, White patients had significantly longer times to surgery than Black patients (p = 0.04); patients with English-speaking caregivers had significantly longer times to surgery than patients with non-English or non-Spanish-speaking caregivers (p = 0.001); and patients on Medicaid had significantly shorter times to surgery than either commercially insured patients (p = 0.02) or patients who were uninsured or self-pay (p < 0.001).

Characteristics	Median time to surgery in days (interquartile range)	P-value
Age	Rho = −0.01	0.88^†^
Gender		0.25^††^
Female	24 (11, 49)	
Male	31 (14, 52)	
Race/ethnicity		0.02*
Asian	27.5 (7, 50)	
Black	19 (10, 43)	
Hispanic	25.5 (11.5, 51)	
White	32 (16, 55)	
Other	33.5 (16, 48)	
Language		<0.001*
English	32 (15, 53)	
Spanish	24 (10, 53)	
Other	16.5 (9, 28)	
Income	Rho = 0.12	0.002
Distance		0.53
<=30 mins	32 (14, 54)	
31-45 mins	27 (12, 54)	
46-105 mins	32 (14, 52)	
>=106 mins	32 (15, 48)	
Insurance status		<0.001*
Commercial	33 (14, 57)	
Medicaid	25 (13, 43)	
Uninsured/self-pay	38 (19, 57)	
Type of surgery		0.11
Endourology	26.5 (13, 52)	
Hydrocele/hernia repair	28 (12, 42)	
Penile surgery	30 (14, 50)	
Testicular surgery	36 (18, 59)	
Ureteral reconstruction	53 (24, 70)	
Other	32.5 (16, 55)	

There was no significant association between time to surgery and median distance to the hospital. There was a significant positive correlation between median income and time to surgery, meaning patients with lower income tended to have shorter times to surgery (p = 0.002). Patients who had no insurance or who were self-pay were found to have the longest median time to surgery (38 days; IQR: 19, 57), and had a significantly longer median time to surgery compared to patients with Medicaid (25 days; IQR: 13, 43) (p < 0.001). Patients with commercial insurance were also found to have longer times to surgery (33 days; IQR: 14, 57) compared to Medicaid patients (p = 0.02). No statistically significant association was found between surgery type and time to surgery (Table 2).

Multiple regression analysis

After adjusting for the other variables in the model, patients whose caregivers spoke languages other than English or Spanish were found to have shorter times to surgery than patients whose caregivers were English speakers (p = 0.03; Table 3). In addition, uninsured patients had longer times to surgery than Medicaid patients (p = 0.01) and commercially insured patients (p = 0.02). Although surgery type was a significant main effect in our model (p = 0.04), no statistically significant differences in time to surgery were found when comparing various surgery types in the post-hoc analysis (Table 3). While patients with higher incomes had slightly longer times to surgery, this was not significant at the 0.05 alpha level (p = 0.05). Age, gender, race/ethnicity, and distance to the clinic were not statistically significant in the adjusted model.

**Table 3:** Model-adjusted geometric means for days to surgery from the linear model with log-transformed days to surgery as the outcome. † Means adjusted for all other variables in this table, which are all in the same model. * Model-adjusted means of log-transformed days to surgery were exponentiated to get geometric means for days to surgery. ^ Age and income (in $10,000s) were continuous variables in the model and for their model-adjusted geometric means, two values one unit apart were chosen to help indicate the direction of the association. Significant Tukey-Kramer-adjusted post-hoc pairwise comparisons for significant variables with three or more groups; language: English-speaking patients had a longer mean log-time to surgery than patients who spoke neither English nor Spanish (p = 0.03); insurance status: uninsured patients had a longer mean log-time to surgery than Medicaid patients (p = 0.01) and commercial patients (p = 0.02).

Categories	Model-adjusted^†^ geometric* mean time to surgery (in days)	95% confidence interval		P-value
Age at visit^				0.26
1 year old	21.2	16.6	27	
2 years old	21.4	16.9	27.1	
Sex				0.34
Female	20.5	14.8	28.3	
Male	23.7	19.2	29.3	
Race/ethnicity				0.13
Asian	19.2	12.8	28.7	
Black	18.4	13.1	25.7	
Hispanic	22.5	17	29.7	
Other	28.1	20.3	39	
White	23.4	18.4	29.8	
Language				0.03
Other	17.4	12.1	25.1	
Spanish	22.8	15.1	34.3	
English	27.1	22.2	33	
Median income^				0.05
$60,000	21.8	17.4	27.2	
$70,000	22.7	18	28.7	
Distance				0.83
<= 30 mins	21.8	17.1	27.9	
31-45 mins	21.2	16.2	27.8	
46-105 mins	23.4	18.1	30.3	
106+ mins	21.8	16.6	28.7	
Insurance				0.01
Medicaid	19.6	15.4	24.9	
Uninsured	26.9	20.4	35.5	
Commercial	20.4	15.9	26	
Surgery type				0.04
Endourology	18.1	14.1	23.1	
Hydrocele/hernia	17.9	13.4	24	
Other	19.7	13.3	29.1	
Testicular surgery	24.9	18.9	32.8	
Ureteral reconstruction	32.3	16.9	61.7	
Penile surgery	22.4	17.7	28.4	

## Discussion

In our analysis, patients with caregivers who spoke languages other than English or Spanish had a shorter time to surgery compared to patients with caregivers who spoke primarily English. The absence of insurance was associated with increased time to surgery, compared to patients on Medicaid. We also found borderline significant associations that patients with lower median household incomes had a shorter time to surgery than higher-income patients.

Many of our findings are contrary to what has been published in the literature regarding health disparities in pediatric specialty care, including significant differences in access to care for racial and ethnic minorities [1-3,5-7,12-16]. Much of the previous literature that documented disparities for racial and ethnic minorities focused on delays or suboptimal care with acute presentations and disease exacerbations including acute appendicitis [5,11], otitis media [12], nephrolithiasis [4], and asthma [3,6]. Previous work conducted by Chu et al. found that Black children had a statistically significant higher odds ratio of having postoperative complications following pediatric urologic procedures [13]. Conversely, multiple studies have documented that non-White children tend to have an earlier surgical intervention for ureteropelvic junction obstruction when presenting postnatally [14-16]. Among their findings, Routh et al. also found that non-White patients were more likely to have surgery in the first year of life. They posited that this might be due to decreased access to prenatal care causing more emergent surgery to be warranted [14]. Although not seen in our linear model, our bivariate analysis aligns with their previous findings that Black patients tend to have a shorter time to surgery intervals than White patients. One possible explanation for this finding is that more of the surgeries being pursued by White patients are deemed elective or less urgent compared to surgeries for non-White patients.

Another unanticipated finding was that patient caregivers with English as a primary language had longer times to surgery compared to patients with caregivers primarily speaking languages other than English or Spanish. Several studies have reported results contrary to ours [20-23]. Steinberg et al. found in their 48 interviews with Spanish-speaking mothers that the majority reported difficult experiences with healthcare staff resulting in barriers to care when accessing specialty care for their children [20]. Doshi et al. found in their interviews with 28 healthcare workers and social service providers that transportation, financial costs, and concerns about language services were some of the most concerning barriers for their patients [21]. In their investigation of pediatric patients with appendicitis and limited English proficiency, Stokes et al. found that patients with limited English proficiency did not encounter delays, but were often discharged without a plan from their pediatrician's office or an emergency setting [23]. While our study finds that being a non-English or non-Spanish speaker was associated with decreased time intervals from the clinic to the operating room, it should be noted that the patients included in our analysis were those that were identified, referred, and underwent surgery. Individuals who were scheduled for surgery but who did not undergo their procedure due to any of the previously mentioned reasons were not included in this study, which may account for this discrepancy from our findings and the literature.

Multiple studies have documented barriers that exist in access to healthcare when patients lack insurance [8-10,12,24-26]. Bjornstad et al. found that children without insurance are more likely to suffer acute kidney injury when admitted to hospitals [24]. Bisgaier and Rhodes found a disparity in access to outpatient specialty care between children with public insurance compared to those with private insurance [9], while Wang et al. found limited access to surgical specialty healthcare for children with government-funded insurance compared to those with commercial insurance in Southern California [25]. We did find that uninsured patients had a longer time to surgery than Medicaid patients. This may be best explained by Loehrer et al., who found that amongst states that expanded Medicaid to encompass larger portions of the population, they noted a decrease in complicated disease presentation and an increase in optimal disease management [26]. Although that study focused on adults, expansion of Medicaid and insurance coverage may encourage young parents to best utilize the services they have available for their children in a timelier fashion. For patients who lack health insurance for their children, parents may be more likely to defer surgery or wait until they have a change in insurance status or the financial means to cover the procedure.

Our final unanticipated finding was that higher socioeconomic status (SES) bordered on the association with longer time intervals to surgery. Researchers in Canada investigating the associations with SES had previously found no association between SES and time intervals to surgery [27,28]. However, this was not found to be true for Rubinger et al. in their assessment of pediatric neurosurgical patients undergoing epilepsy surgery. They found that the lowest SES patients had a longer time to surgery and often had higher seizure frequency following the procedures than their more affluent counterparts [29]. A possible explanation is that many urologic procedures are elective in nature, and can thus be planned out with more anticipation by more affluent parents creating a longer time to surgery.

To our knowledge, this is the first study to analyze health disparities in pediatric patients undergoing urologic surgery regarding time to surgical intervention. As the first study of its kind, it includes patients from a wide variety of socioeconomic and demographic backgrounds. Our study’s strengths include both the sample size and the number of variables analyzed in our analysis. Our population sample is diverse in the representation of race and ethnicity and closely mirrors our community’s racial and ethnic diversity. Our sample population's racial and ethnic demographics compared to our community demographics were, respectively, White (64.8-66.6%), Black (9.3-12.3%), Hispanic (13.2-13.9%), and Asian (4.3-3.8%) [30]. It also includes patients from both rural and urban backgrounds, as the catchment of our institution serves patients from all over the surrounding region, evidenced by 50.2% of our sample traveling 46 minutes or more to receive care.

Our data have several limitations related to the study design. One such limitation is the utilization of retrospectively collected chart data from a single institution. Patients may have missing data, may have been lost to follow-up after their recommendation for surgical intervention, or may have scheduled surgery at an outside facility, and we are unable to account for these missing data. Furthermore, we were unable to capture information on surgeries that may have been delayed, including reasons for their delay (e.g. planning surgeries around school breaks). Variables such as income and distance to the clinic were estimated using summary data at the zip code level, which might not reflect individual patients. In addition, our results may not be generalizable to other pediatric populations. As a single institution serving a largely rural state, our patient demographics may not be comparable to those of other children’s hospitals.

To better understand the factors and barriers affecting pediatric urologic care, further research in pediatric urologic surgery at multiple institutions is needed. A reinvestigation utilizing similar variables with a larger sample size and across a larger geographic area may help better understand variation in access to care and healthcare disparities among patients needing pediatric urologic surgery.

## Conclusions

In our patient population, we identified patient demographic factors associated with increases in time from initial evaluation to surgical intervention among pediatric patients undergoing urologic surgery including primary language spoken and insurance status. Additional research is needed to better understand variations in access to pediatric urologic surgery.
